# Densitometric Quantification and Optimization of Polyphenols in *Phyllanthus maderaspatensis* by HPTLC

**DOI:** 10.1016/j.sjbs.2021.11.019

**Published:** 2021-11-22

**Authors:** UK Ilyas, Muhammed Elayadeth-Meethal, Mohamed Saheer Kuruniyan, Syed Altafuddin Quadri, R.S. Rajasree, Punnoth Poonkuzhi Naseef

**Affiliations:** a Department of Pharmacognosy and Phytochemistry, Moulana College of Pharmacy, Perinthalmanna, 679321, Kerala, India; b Department of Animal Breeding and Genetics, Kerala Veterinary and Animal Sciences University, Wayanad, 673576, Kerala, India; c Department of Dental Technology, College of Applied Medical Sciences, King Khalid University, Abha 61421, Saudi Arabia; d College of Pharmaceutical Sciences, Government Thirumala Devaswom Medical College, Alappuzha 688005, India; e Department of Pharmaceutics, Moulana College of Pharmacy, Perinthalmanna, 679321, Kerala, India

**Keywords:** Polyphenols, Purification, Extraction, Chromatography, Validated HPTLC, Box-Behnken design, Phyllanthus maderaspatensis

## Abstract

Quantifying and optimizing the polyphenol content of *Phyllanthus maderaspatensis* was accomplished using a single-solvent HPTLC system. Analyzing hydroalcoholic extracts for kaempferol, rutin, ellagic acid, quercetin, catechin, and gallic acid, we simultaneously quantified and optimized their concentration. In the experiment, the methanol to water ratio (%), temperature (°C), and time of extraction (min) were all optimized using a Box-Behnken statistical design. Kaempferol, rutin, ellagic acid, quercetin, catechin, and gallic acid were among the dependent variables analyzed. In the HPTLC separation, silica gel 60F254 plates were used, and toluene, ethyl acetate, and formic acid (5:4:1) made up the mobile phase. For kaempferol, rutin, ellagic acid, quercetin, catechin, and gallic acid, densitometric measurements were carried out using the absorbance mode at 254 nm. Hydroalcoholic extract of *P. maderaspatensis* contains rutin (0.344), catechin (2.62), gallic acid (0.93), ellagic acid (0.172), quercetin (0.0108) and kaempferol (0.06). Further, it may be affected by more than one factor at a time, resulting in a varying degree of reaction. A negative correlation was found between X1 (extraction time (min)) and X2 (temperature), as well as X1 and X3 (solvent ratios). Taking these characteristics into consideration, the method outlined here is a validated HPTLC method for measuring kaempferol, rutin, ellagic acid, quercetin, catechin, and gallic acid.

## 1 Introduction

Herbal plants of the Phyllanthus family (*Euphorbiaceae*) such as *Phyllanthus maderaspatensis* grow in southern India, China, Sri Lanka, and South Africa. Hepatoprotective properties make it a traditional remedy in India. Herbs and fruits contain polyphenolic compounds that exhibit multiple beneficial physiological effects, such as antiviral, antibacterial, vasodilatory, antioxidant, anti-inflammatory, and antiradical effects (Bacchetti et al., 2020). As well as this they also improve memory, promote oral health, are antioxidative, anti-inflammatory, anticancer, provide cardiovascular protection, offer gastroprotection, and regulate the immune system (Uddin et al., 2020, Yang and Zhang, 2018, Ginwala et al., 2019, Basu et al., 2018, Campos-Vidal et al., 2021, Magrone et al., 2020). By inhibiting apoptosis and preventing tumor-angiogenesis it is also effective in preventing the development and metastasis of various cancers (García-García et al., 2021) Additionally, they exert chemo preventive effects (Mohan et al., 2013).

Humans are considered to benefit from the polyphenolic compounds found in green tea, called catechins (Patil and Balaraman, 2011). Besides antioxidant properties, it is also antibacterial, anticancer, antiradical, and antiviral (Vilkickyte et al., 2020). There is evidence that catechins are effective in the treatment of cardiovascular conditions, cardiomyocyte injury, spermatogenic disorders, brain toxicity, carbonyl reductase1 inhibition, and cancer (Zheng et al., 2011, Choy et al., 2019, Bimonte et al., 2019, Opuwari and Monsees, 2020, Evtyugin et al., 2020).

Pomegranates and strawberries, for example, contain ellagic acid, a naturally occurring phenolic compound. Even at high concentrations ellagic acid is non-toxic and has a wide range of health benefits (Casedas et al., 2020). The medication is used as first-line therapy to prevent liver damage during tuberculosis treatment (Ambrose et al., 2013). Human low-density lipoprotein (LDL) oxidation has been slowed by ellagic acid, catechin, and caffeic acid, which are antioxidative and antiestrogenic, respectively (Olivas-Aguirre et al., 2020). Furthermore, indomethacin-induced gastric ulcer healing is also reported (Aslan et al., 2020).

In addition to their anti-inflammatory properties, gallic acid, catechin, and epicatechin are antineoplastic, antiasthmatic, and radioprotective (Shahidi and Yeo, 2018). The availability of flavonoid glycosides and multi-potent bioflavonoids in natural products tends to exceed the availability of flavonoids (Sharifi-Rad et al., 2020). There are anti-inflammatory, antineoplastic, and immunomodulatory properties for quercetin (Lesjak et al., 2018, Cheng et al., 2019). Rutin is a non-toxic flavonoid that appears in many plants (Gullon et al., 2017). The rutinoside moiety is located at position 3 of the C-ring and the aglycone quercetin is located at position 2 (Nile and Park, 2015). Several of antioxidant, free-radical scavenging, and chemoprevention actions of rutinhave been demonstrated (Ilyas et al., 2021).

HPTLC offers many advantages, such as simplicity, accuracy, cost management, and rapid results, making it an alternative to HPLC that is useful for estimating the active ingredients in herbs (Bucciantini et al., 2021). Some of the advantage of HPTLC are ability to analyse extracts containing multi-active constituents in which many samples can be distinguished parallel to each other on the same HPTLC (Vasilisa et al., 2020). Vasilisa et al. (2020) also established the mobile phase mixture of diethyl ether, formic acid, acetic acid, water, acetophenone and heptane (30:3:9:50:30:10) (v/v/v/v/v/v) [29]. Ilyas et al., 2015a, Ilyas et al., 2015b) reported a solvent mixture of toluene: ethyl acetate, formic acid and methanol (3:3:0.8:0.2) (v/v/v/v) respectively, for quantification of polyphenols. Recently, Haida et al (2021) determined significant extraction factors for extracting polyphenols from lemon using two-level factorial design. The present study therefore used Box-Behnken-assisted statistical methods to investigate the occurrence of polyphenols in *P. maderaspatensis*. Apparently this is the first time polyphenols from *P. maderaspatensis* have been studied and optimized simultaneously in the mobile mixture of toluene, ethyl acetate and formic acid (4:3:1) (v/v/v) that provides good resolution of the peak associated biomarkers from those of closely related compounds in *P. maderaspatensis.*

## 2 Materials and methods

### 2.1 Chemicals and reagents

Among the products purchased from Natural Remedies Pvt. Ltd, Bangalore, India, were kaempferol, rutin, ellagic acid, quercetin, catechin, and gallic acid.SD Fine Chemicals (Mumbai, India) supplied us with methanol (HPLC grade). We purchased toluene, ethyl acetate, and formic acid from CDH Labs (Mumbai, India). 2- amino ethyl diphenylborinate was provided by Sigma Aldrich LLC (St. Louis, MO, USA). Before use, all solutions were filtered through syringe-driven filters of 0.22 μm (HIMEDIA, Mumbai-India).

### 2.2 Equipment used

To make plant extracts by hot percolation, a Soxhlet extractor (Omega, Mumbai, India) was used. The extracts were dried under vacuum using a rotary evaporator (Buchi R-114, Switzerland). HPTLC analysis of polyphenols was performed using a CAMAG HPTLC system equipped with a Linomat IV sample applicator (Muttenz, Switzerland). Applying extracts was performed using pre-coated silica gel 60F254 (Merck, Germany)-backed aluminum TLC plates (20 × 10 cm). We developed TLC plates by vertical development using CAMAG twin trough chambers.

### 2.3 Plant material collection

We collected the leaves from Maruthmallai, Kanyakumari district, Tamilnadu in February 2011. Dr. V. Chelladurai, Research Officer, Central Council for Research in Ayurveda and Siddha (Govt. of India), Tirunelveli, Tamil Nadu, identified and authenticated it.

### 2.4 Plant material extraction

First, we dried and powdered plant materialthen extracted it in 95% ethanol and 50% hydro alcohol for 6 h at 37 °C. A three-time extraction procedure was used. Combining ethanolic and hydro-alcoholic extracts resulted in the desired result. To evaporate the liquid to dryness, the 40 °C temperature was set on an evaporator under reduced pressure. In the extract prepared with 95% ethanol, the yield was 14.98% weight-for-weight, whereas the extract prepared with hydro-alcoholic alcohol yielded 15.58% weight-for-weight. Using dried plant material, the aqueous extract was prepared by boiling it ten times with distilled water for two hours at 60–70 °C. Filtered and evaporated decoctions then went into a water bath at 50–60 °C to evaporate additional components.

### 2.5 Preparation of sample solutions

The 100 mg of crude extract was dissolved in 10 ml of methanol (HPLC grade), sonicated for about 10 min, then filtered through a syringe filter (0.22 μm) and injected with HPTLC.

### 2.6 Calibrating standard markers according to a calibration curve

Three triplicates of kaempferol, rutin, ellagic acid, quercetin, catechin, and gallic acid were spotted using a CAMMAG Linomat-5 sample spotter attached to silica gel 60 F254 plates. The plates were developed in a CAMAG 20 * 10 cm twin trough chamber (20:4:1) using toluene, ethyl acetate, and formic acid (5:4:1). Following air drying, the plates were scanned using a CAMAG TLC scanner with winCATS 4 at 366 nm. An area of peak activity was measured. Using peak areas concerning applied crude extract concentrations of kaempferol, rutin, ellagic acid, quercetin, catechin, and gallic acid, calibration curves were constructed for these compounds.

### 2.7 Analyzing various markers in extracts of *P. maderaspatensis*

Three spots each of 10 µl of sample solution (each) were spotted on silica gel 60 F254 plates using an automatic sample spotter from CAMAG. It was possible to measure peak area and absorption spectrum. Based on the respective standard calibration curves of kaempferol, rutin, ellagic acid, quercetin, catechin, and gallic acid, bioactive compounds were determined in *P. maderaspatensis*.

### 2.8 Percentage yield of polyphenols

Firstly, the plant (100 g) and the extracts (15.58 %w/w) should be dried completely (no any trace of moisture and solvent remain). The percentage yield was calculated from the following equation:

Yield%=weight of the dry extract x100/weight of the dry plant.

The 100 mg of crude extract was dissolved in 10 ml of methanol (HPLC grade), sonicated for about 10 min, then filtered through a syringe filter (0.22 μm) and injected with HPTLC. Calibration curve of Rutin, catechin, gallic acid, ellagic acid, quercetin, and kaempferol (100–1600, 200–1400, 100–1000, 40–140, 60–600, and 40–200 ng/band) were obtained by plotting peak areas versus applied crude extract (10 µL). 10 µL each of sample solutions were applied in triplicate on silica gel 60 F_254_ plates with CAMAG Linomat-5 Automatic Sample Spotter. The peak areas and absorption spectra were recorded. The amount of polyphenols in of *P. maderaspatensis* was calculated using the respective standard calibration curves.

A method for optimizing polyphenol content from hydroalcoholic extract

Using the Box-Behnken statistical design, three factors and levels were considered. There were seventeen runs of it. To optimize the design, the Stat-Ease V6 software (Minneapolis, MN, USA) was used. This approach may be used in analyzing quadratic response surfaces and for the creation of second-order polynomial models. Plots arranged in groups at the center of each edge were used in the experiment. Additionally, the four-dimensional cube had a replicated center point, which defined the region of interest. Table 1 provides information on both the independent variables and the dependent variables.

**Table 1:** Independent factors and selected levels.

**Independent variables**	**Levels**		
	**Low (-1)**	**Medium**	**High (+1)**
X_1_ = Time in min	30	60	90
X_2_ = Temperature (°C)	30	45	60
X_3_ = Solvent ratio (v/v)	40	60	80
**Dependent variables**	**Goals**		
Y_1_ = Rutin	Maximized		
Y_2_ = Kaempferol	Maximized		
Y_3_ = Ellagic acid	Maximized		
Y_4_ = Quercetin	Maximized		
Y_5_ = Catechin	Maximized		

Following is the polynomial equation generated by the experimental design.

R = C0 + C1 X1 + C2 X2 + C3 X3 + C4 X1 X2 + C5 X1 X3 + C6 X2 X3 + C7 X12 + C8 X22 + C9 X32.

X1 denotes the dependent variable, X2, and X3 denote the independent variables. C0 indicates the intercept, C1 to C9 represents the regression coefficients, and R represents the dependent variable. Table 2 shows the detailed experimental style. The data were analyzed using y the Design-Expert software system (See Table 3).

**Table 2:** Experimental runs and their observed responses.

Independent variables				Dependent variables				
Run	Factor-1 (X_1_) Time (Min)	Factor-2 (X_2_) Temp. (°C)	Factor-3 (X_3_) Solvent ratio	Rutin (Y_1_)	Kaempferol (Y_2_)	Ellagic acid (Y_3_)	Quercetin (Y_4_)	Catechin (Y_5_)
01	30	30	60	0.007	0.06	0.063	0.005	0.51
02	90	30	60	0.015	0.049	1.3	0.019	0.15
03	30	60	60	0.009	0.036	1.4	0.0008	0.4
04	90	60	60	0.01	0.083	0.97	0.061	0.48
05	30	45	40	0.044	0.0045	0.71	0.06	0.37
06	90	45	40	0.09	0.099	1.97	0.06	0.37
07	30	45	80	0.4	0.086	1.7	0.043	0.42
08	90	45	80	0.35	0.034	0.69	0.12	0.16
09	60	30	40	0.42	0.0002	0.02	0.07	0.02
10	60	60	40	0.33	0.103	1.13	0.08	0.2
11	60	30	60	0.64	0.104	0.78	0.08	0.03
12	60	60	80	0.73	0.0156	0.10	0.11	0.016
13	60	45	60	0.84	0.056	1.05	0.082	0.00
14	60	45	60	0.84	0.055	1.05	0.082	0.00
15	60	45	60	0.85	0.0556	1.05	0.081	0.00
16	60	45	60	0.84	0.0556	1.05	0.080	0.00
17	60	45	60	0.83	0.0556	1.05	0.082	0.00

**Table 3:** Quantification parameters used for the polyphenols by HPTLC method.

Parameters	Rutin	Catechin	Gallic acid	Ellagic acid	Quercetin	Kaempferol
Linearity Range (ng)	100–1600	200–1400	100–5000	20–200	10–160	40–200
Correlation coefficient	0.996	0.999	0.9972	0.9993	0.9996	0.992
Regression equation (ng/band)	Y = 11.1 + 4.4 X	Y = 3.8 + 103.5X	Y = 2292.8 + 3.3 X	Y = -204.7 + 25.4 X	Y = -149.5 + 82.2 X	Y = 2.9 + 0.91X
Rf values	0.08	0.52	0.55	0.53	0.62	0.68
Alcoholic extract (% w/w)	0.0198	Nil	0.26	Nil	0.035	0.015
Aqueous alcoholic (% w/w)	0.344	0.262	0.93	0.172	0.108	0.06
Decoctions (% w/w)	0.0013	Nil	1.02	Nil	Nil	Nil

## 3 Results

### 3.1 *P. maderaspatensis* crude extract yield (in percentages)

There were 15.18 weights per weight for crude ethanolic extract, 15.58 weights per weight for ethanolic aqueous extract, and 13.7 weights per weight for aqueous extract, respectively.

### 3.2 Mobile phase optimization

Polyphenolic compounds (1 mg/ml) were used for chromatographic separation in methanol with a standard solution. Different solvent systems were initially used. However, the maximum resolution was achieved with a 5:4:1 ethyl acetate/formic acid solution. The Rf values obtained were 0.08 for rutin, 0.52 for catechin, 0.55 for gallic acid, 0.57 for ellagic acid, 0.62 for quercetin, and 0.67 for kaempferol.

**Fig. 1:**
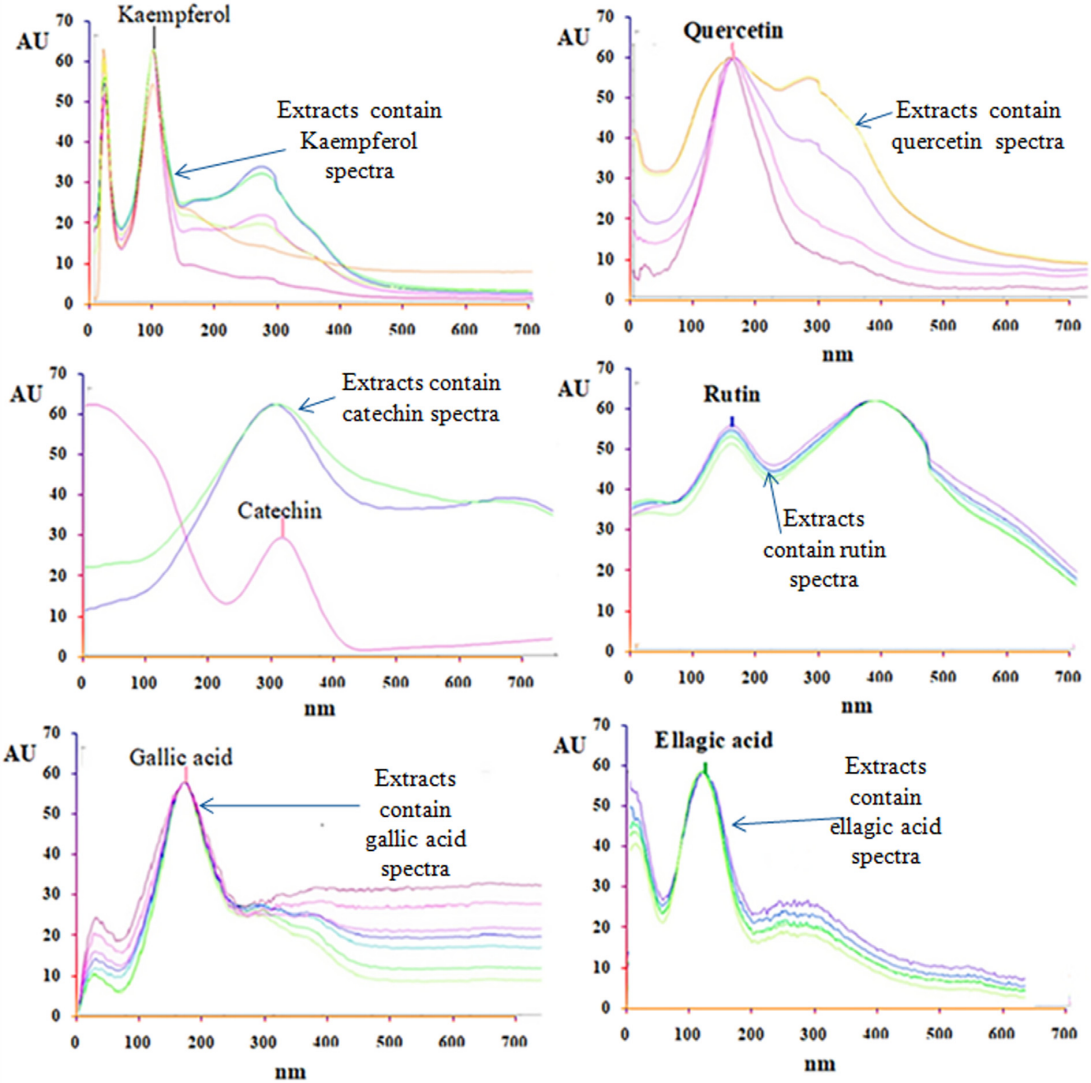
Overlay absorption spectra of various concentrations of standard (kaempferol, quercetin, catechin, gallic acid, ellagic acid) and along with sample extracts containing individual polyphenols spectra.

**Fig. 2:**
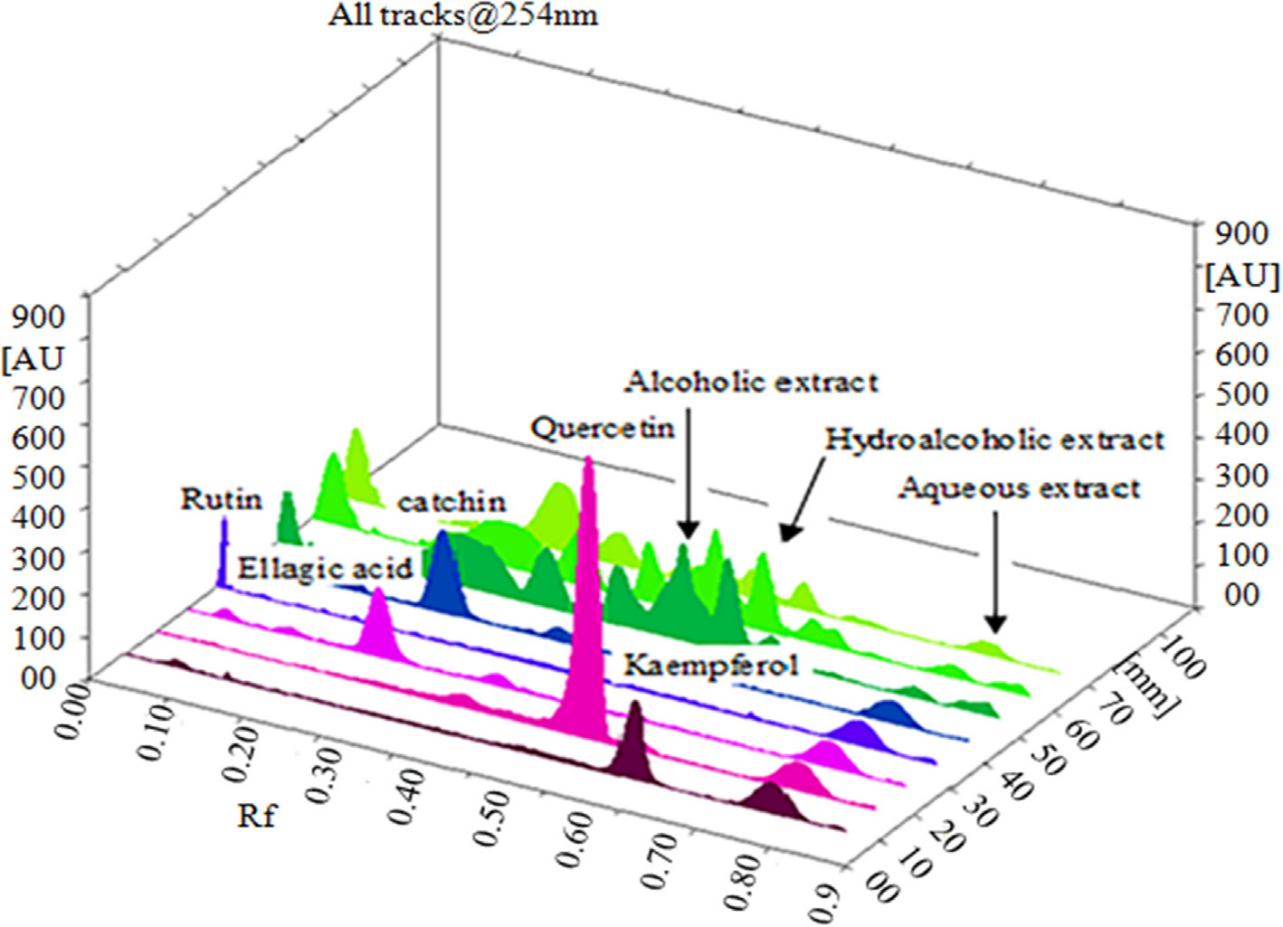
The diagram shows the standard peaks corresponding to the test sample peak. The plate was scanned by the CAMAG TLC scanner 3 for three-dimensional views of all tracks at 254 m.

Then, the optimization and quantification were performed simultaneously. Through overlapping the UV absorption spectra of kaempferol, rutin, ellagic acid, quercetin, catechin, and gallic acid with that of the respective references (Fig. 1), we were able to identify these compounds. Our analysis of the absorption spectrum at the start, center, and end of each band helped determine its purity. In Fig. 2, we show standard peaks corresponding to the test sample peak, corresponding to all tracks scanned with the CAMAG TLC scanner 3 at 254 m (Fig. 2).

Rutin, catechin, gallic acid, ellagic acid, quercetin, and kaempferol showed linearities of 100–1600, 200–1400, 100–1000, 40–140, 60–600, and 40–200 ng/band, and the correlation coefficients for these variables were 0.996, 0.999, 0.9993, 0.9996, and 0.992, respectively.

### 3.3 Statistical design by Box Behnken

In this Box-Behnken study, 17 trials were conducted. Three factors were assessed along with three levels. The independent factors showed different responses as indicated in Table 2. Using the quadratic model, we were able to determine the concentrations of the compounds kaempferol, rutin, ellagic acid, quercetin, catechin, and gallic acid. The table below displays a comparison of the selected responses of this report based on R, SD, percent C.V, and regression equations. For each of the following compounds, equations were derived using their statistically significant values (p < 0.0007), rutin (p < 0.0001), catechin (p < 0.0012), ellagic acid (p < 0.0001), and quercetin (p < 0.0001). For factors that support optimization, positive values were assigned, while for factors that weaken optimization, negative values were assigned.

In the study, the factors time of extraction (min, X1) and temperature (X2) had a negative impact. For the solvent ratio (X3), however, a positive effect was obtained for all five responses. Results show that the response and variables have a non-linear relationship. The degree of difference in response was observed when more than one factor was changed simultaneously. All selected responses were negatively impacted by the interaction between the variables X1 and X2, as well as between X1 and X3. Different factors had varying square roots, however. A positive impact was shown by factors X22 and X32, while a negative effect was demonstrated by factor X12 (Table 4). Final composition ratios of extractions were determined based on the percentage yields of polyphenols.

**Table 4:** Summary of regression analysis for different models and responses (Y1 toY5).

Rutin (Y_1_)					
Models F value	*R^2^*	Adjusted *R_2_*	Predicted *R_2_*	SD	C.V.%
Linear	0.0956	0.1130	−0.4741	0.37	–
2F_1_	0.1007	0.4389	−1.8788	0.47	–
Cubic	0.9938	0.9858	–	0.042	–
Quadratic	0.9984	0.9936	0.9236	0.028	9.81
Catechin (I)					
Linear	0.1065	0.0997	0.6294	0.21	–
2F_1_	0.2275	0.2360	1.9675	0.23	–
Cubic	1.000	1.000	–	0.000	–
Quadratic	0.9450	0.8743	0.1203	0.073	39.65
Kaempferol (Y_3_)					
Linear	0.0599	−0.1570	−0.8741	0.037	–
2F_1_	0.8658	0.7852	0.6098	0.016	–
Cubic	0.9611	0.7969	–	0.015	–
Quadratic	0.9285	0.7141	0.7000	0.018	28.59
Quercetin (Y_4_)					
Linear	0.2479	0.0744	0.5003	0.032	–
2F_1_	0.3904	0.0246	1.8245	0.032	–
Cubic	0.9995	0.9980	–	0.0015	–
Quadratic	0.9973	0.9939	0.9648	0.0026	3.89
Ellagic acid (Y_5_)					
Linear	0.0425	0.1785	0.9959	0.53	–
2F_1_	0.6571	0.4519	0.7263	0.36	–
Cubic	0.9974	0.9914	–	0.046	–
Quadratic	0.9897	0.9765	0.8669	0.075	7.67

#### 3.3.1 Independent variables and the percentage yield of rutin (Y1)

Y1 = − 4.38 + 0.078 X1 + 0.094 X2 + 0.0175 X3 − 0.000004 X1 X2 − 0.000035 X1 X3 + 0.00015 X2 X3 − 0.000631 X12 − 0.00114 X22 − 0.00012 X32

**Fig. 3:**
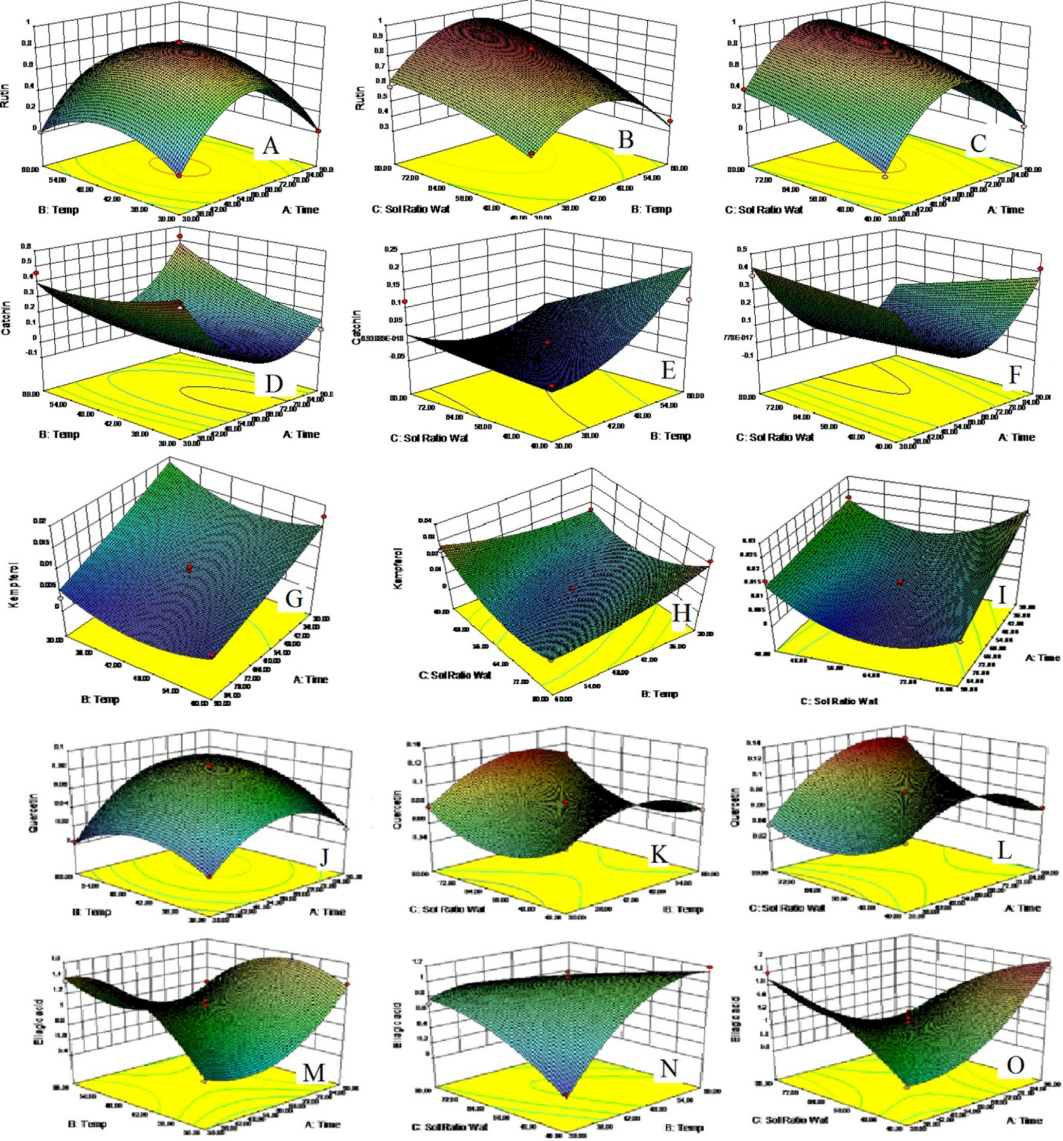
Response surface plots A, D, G, J, and M shows response surface plots of factor X2 vs. X1 against polyphenols (rutin (Y1), catechin (Y2), kaempferol (Y3), quercetin (Y4) and ellagic acid (Y7) respectively); B, E, H, K, and N shows response surface plots of factor X3 vs. X2 against polyphenols; C, F, I, L, and O show response surface plots of factor X3 vs. X1 against polyphenols.

Y1 represents polyphenol yield in percentage, X1 represents time of extraction in minutes, X2 represents extraction temperature in °C, and X3 represents the required solvent ratio (methanol/water v/v) to extract the maximum amount of rutin. By extending the extraction time, we obtained a higher percentage yield of rutin. However, it decreased after an optimum value possibly due to the saturation of the compound. The X2 also showed positive effects on the percentage yield of rutin. Because the compound is thermostable, its percentage yield increases as the temperature increases. The percentage yield of rutin decreased after the temperature reached an optimum value as a result of degradation (Fig. 3B). The Rutin ratio (X3) increased with the increasing solvent ratio. When the solvent ratio was higher, the rutin percentage yield was greater (Fig. 3C). This may be due to the compound's polarity.

#### 3.3.2 Catechin (Y2) yield influenced by independent factors

Y2 = 0.0–0.066 X1 + 0.055 X2 − 0.039 X3 + 0.11 X1 X2 − 0.068 X1 X3

+ 0.06 X2 X3 + 0.33 X12 + 0.056 X22 + 0.0004 X32

In comparison with X2, X1 negatively affected the percentage yield of catechin. When we enhanced the extraction time it caused a reduction in the percentage yield of catechin, possibly due to the poor penetration of solvent as shown in Fig. 3D.

Since catechins possess a high degree of stability (Fig. 3E), an increase in temperature increased their percentage yield.

The percentage yield of catechin was negatively affected by factor X3. This might be because the compound has a medium polarity which results in a decrease in percentage yield at higher solvent ratios. As can be seen in Fig. 3F, different solvent ratios result in different catechin yields.

#### 3.3.3 Kaempferol (Y3) percentage yield influenced by independent factors

Y3 = 0.056 + 0.11 X1 + 0.0038 X2 + 0.0044 X3 + 0.013 X1 X2 − 0.037 X1 X3 − 0.048 X2 X3

A change in kaempferol yield was observed with Factor X1, as observed with Factor X2. The percentage yield of kaempferol increased upon increasing the time of extraction due to the attainment of optimum penetration time (Fig. 3G).

Compared with factors X1, time of extraction (min), and X2, extraction temperature, factor X2 did not significantly affect the percentage yield of kaempferol (Fig. 3H).

As a result of a lesser penetration rate, factor X3, the solvent ratio, had significantly less effect on percent kaempferol yield compared to factor X2 (Fig. 3I).

#### 3.3.4 Percentage yield of quercetin (Y4) as a function of independent factors

Y4 = 0.082 + 0.091 X1 + 0.0009 X2 + 0.001 X3 + 0.012 X1 X2 + 0.021 X1 X3 + 0.0007 X2 X3 −0.036 X12 − 0.025 X22 + 0.026 X32

Factors X1 and X2 both had the same influence on the percentage yield of quercetin. The percentage yield of quercetin grew as the extraction time was extended until an optimum period, after which it declined, probably due to the polar character of the compound as demonstrated in (Fig. 3J). When compared to variables X1 and X3, factor X2 had a less pronounced effect on percentage yield. Because of the thermo-labile nature of quercetin, the percentage yield of quercetin grew slowly with increasing temperature until a certain point, after which it declined. The influence of temperature on quercetin percentage yield is shown in Fig. 3K. As compared to temperature, factor X3 had a less favorable effect on the percentage yield of quercetin. Because of the poor solvent penetration, an increase in the solvent ratio had no meaningful effect on percentage yield. Fig. 3L depicts the influence of different solvent ratios on the percentage yield of quercetin.

#### 3.3.5 Effects of independent variables on ellagic acid yield percentage (Y5)

Y5 = 1.05 + 0.061 X1 + 0.11 X2 + 0.069 X3 − 0.28 X1 X2 − 0.57 X1 X3 − 0.45 X2 X3 + 0.39 X12 −0.37 X22 −0.18 X32

The percentage yield of ellagic acid has been changed by factor X1 indicating extraction time (min). It was observed that when the extraction time was extended, the percentage yield of ellagic acid first dropped. However, it surged within a short period due to the high solvent penetration level, as demonstrated in (Fig. 3M). The influence of the temperature on ellagic acid percentage yield is greater with factor X2 than factor X1. A thermostable compound, ellagic acid increased drastically in percentage yield with temperature. As shown in figure (Fig. 3N), it decreased slowly after the optimum temperature, which may be caused by the degradation of the compound. The ellagic acid yield was markedly positively affected by factor X3. A higher ratio of solvent promoting ellagic acid also resulted in a higher percent yield of ellagic acid as a result of its polar nature (Fig. 3O).

### 3.4 Identification of polyphenols

Kaempferol: UV spectra of isolated compound showed 366 nm, the fundamental structure of isolated compounds appeared as like flavonoids was confirmed by TLC derivatization with NP reagents. It absolutely was characterized as rutin when matching with authentic sample of rutin by co-TLC. The EI-MS *m*/*z* 287.055 (M + H) ^+^; 285.141 (M−H), 227, 159 indicated that kaempferol was the compound.

Rutin: Orange colour with NP-PEG reagent at 366 nm. It was characterized as rutin when matching with authentic sample of rutin by co-TLC. Mass spectral studies revealed a sharp peak with an *m*/*z* value 611.1 which corresponds to the molecular weight of Rutin (610.2). The EI-MS *m*/*z* 611.1 (M + H) ^+^; 609.2050 (M−H)^-^; 300.128, 271.103, 227.1, 151.01, Based on MS, the major peak was confirmed to be that of rutin.

Ellagic acid: UV λmax (MeOH): 290 nm. It was characterized as ellagic acid when matching with authentic sample of ellagic acid by co-TLC EI-MS *m*/*z* 303.391(M + H)^+^; 301.234 (M−H)^-^; 283, 271.9, 256.8, 228.9 (100%), 212.9, 200.9 and 184.9. As per the data in hand and available in literature, the compound was characterized as ellagic acid.

Quercetin: Florescence yellow colour was obtained with NP-PEG reagent at 366 nm. It was characterized as quercetin when matching with authentic sample of quercetin by co-TLC. The EI-MS revealed with *m*/*z* 303.049 ([M + H]); 301.036 ([M−H]), 179, 151. Based on MS, the major peak was confirmed to be that of quercetin.

Catechin: Basic structure of isolated compound appeared as like flavanoids was confirmed by TLC spraying with NP reagent. Mass spectral studies revealed a sharp peak with an M/z 291.32 ([M + H]); 289.1422([M−H]), 245.12, 271.1, 203.11(100%), 161, 187 indicated that catechin was the compound.

Gallic acid: grey blue colour was obtained with NP-PEG (natural product and polyethylene glycol) reagent (Sigma). UV λ_max_ (MeOH): 290 nm. solid. UV λmax (MeOH): 290 nm. The EI-MS *m*/*z* 169.11(M−H)^-^. As per the data in hand and available in literature, the compound was characterized as gallic acid.

## 4 Discussion

According to the results, the ethanol in water extract, the crude ethanol extract, and the water extract, yielded 15.18, 15.58, and 13.7 w/w, respectively. A water-based alcoholic extract is more productive than an ethanolic extract since it contains polar water-soluble compounds and polyphenols are more readily soluble in water-based alcohols. Polyphenols are abundant in these aqueous ethanolic extracts when measured as a percentage yield. The study found that the yield of crude ethanolic extract, aqueous ethanolic extract, and aqueous extract was: 15.18 w/w.15.58 w/w, and 13.7 w/w, respectively. The aqueous- ethanolic extract showed a higher yield followed by ethanolic extract due to the presence of water-soluble polar compounds and higher solubility of polyphenols in aqueous alcoholic extracts. The percentage yield indicated that the aqueous ethanolic extracts are a rich source of polyphenols.

The different mobile phases reported previously for the simultaneous separation and quantification of polyphenols were examined by HPTLC technique, namely toluene: ethyl acetate: formic acid: methanol (3:3:0.8:0.2 v/v) and ethyl acetate: formic acid: acetic acid: water (20: 3: 1: 2) (Nile et al.., 2015; Ilyas et al., 2021). Using a newly developed mobile phase containing toluene, ethyl acetate, and formic acid (4: 3: 1), polyphenol quantification and optimization could be achieved simultaneously.

The separation of closely related compounds associated with biomarkers has been demonstrated correctly for the first time using *P. maderaspatensis*. HPTLC data showed that the aqueous alcohol extract contained higher levels of kaempferol, quercetin, catechin, rutin, and ellagic acid than other extracts. Research has consistently shown that long-term consumption of a diet high in plant polyphenols protects against neurodegenerative disorders, cancer, cardiovascular diseases, diabetes, and osteoporosis (Cory et al., 2018, López-Fernández et al., 2020).

The effectiveness of polyphenols depends largely on the extraction and quantification techniques as well as molecules containing polyphenols such as extracts and plant materials (Kumar and Goel, 2019, Krakowska-Sieprawska et al., 2020, Malathy et al., 2021). Polyphenols from plants such as herbs, oils, teas, and fruits have also been extracted from samples by dipping them in extraction solvents (Ilyas et al., 2015a, Ilyas et al., 2015b, Suleria et al., 2020, Cordoba et al., 2019). Several factors contribute to the effectiveness of polyphenol extraction, including chemical composition, temperature, extraction time (minutes), and pH (Pandey and Rizvi, 2009, Juszczak et al., 2019, Alara et al., 2021). As there are so many phenolic compounds present and their quantification methods are so old, there is no common extraction method for all types of polyphenols (Dai and Mumper, 2010, Brglez Mojzer et al., 2016). It is, therefore, possible and valuable to develop and apply new extraction techniques that enable us to accurately optimize polyphenol extraction based on extraction time (minutes), temperature (° C), and solvent ratio (%v/v) incorporating Box Behnken statistical design experts (Patzold et al., 2019, Jamioł et al., 2021, Rifna et al., 2021).

## 5 Conclusions

Herbs and fruits contain polyphenolic compounds. HPTLC was used to extract the polyphenols simultaneously from *P. maderaspatensis* hydroalcoholic extract by a medium solvent system. Extraction variables included time (min), temperature (°C), and the ratio of methanol to water (% v/v). With the Box-Behnken statistical design, we extracted and optimized kaempferol, rutin, ellagic acid, quercetin, catechin, and gallic acid. Maximum percentage yields were obtained with aqueous ethanolic extracts.

## Author Contributions

**U K Ilyas:** Conceptualization, data curation, visualization, writing—original draft preparation and funding acquisition. **R.S. Rajasree:** writing—original draft preparation, writing—review and editing. **Punnoth Poonkuzhi Naseef:** data curation, writing—original draft preparation, writing—review and editing, visualization, supervision and funding acquisition. **Mohamed Saheer Kuruniyan:** Conceptualization, writing—review and editing, funding acquisition. **Muhammed Elayadeth-Meethal:** Conceptualization, data curation, writing—review and editing, visualization. **Syed Altafuddin Quadri:** funding acquisition, writing—review and editing.

## Funding

Authors would like to thank the National Medicinal Plant Board, Department of AYUH, Delhi, India.

## Declaration of Competing Interest

The authors declare that they have no known competing financial interests or personal relationships that could have appeared to influence the work reported in this paper.
